# A Comparison of Methods for Identifying Informal Carers: Self-Declaration Versus a Time Diary

**DOI:** 10.1007/s40273-022-01136-8

**Published:** 2022-04-08

**Authors:** Sean Urwin, Yiu-Shing Lau, Gunn Grande, Matt Sutton

**Affiliations:** 1 Health Organisation, Policy and Economics, School of Health Sciences, University of Manchester, 6th Floor Williamson building, Oxford Road, Manchester, M13 9PL UK; 2 Division of Nursing, Midwifery and Social Work, Manchester Academic Health Science Centre, University of Manchester, Manchester, UK; 3 Melbourne Institute, Applied Economic and Social Research, University of Melbourne, Melbourne, Australia

## Abstract

**Objectives:**

Two main methods for identifying whether an individual is an informal carer are self-declaration and the use of a time diary. We analysed the level and predictors of agreement between these two methods among co-residential informal carers of adult recipients.

**Methods:**

We used the 2014/15 UK Time Use Survey, which is a large-scale household survey for those aged 8 years old and over. It contains an individual questionnaire for self-declaration and a time diary for activity-based identification that records all activity in 10-min slots for two 24-h periods. Our analysis: (i) assesses the degree of overlap across approaches; (ii) explores the differences in characteristics between carers identified via one approach relative to non-carers using a bivariate probit estimator; and (iii) shows what factors are associated with being identified by both approaches using two independent probit estimators.

**Results:**

Out of 6301 individuals, we identified 545 carers (8.6%) by at least one method and only 104 (19.1% of 545 carers) by both methods. We found similar factors predicted caregiving using either method but the magnitudes of the effects of these factors were larger for self-declared carers. Activity-based carers who provided more activities to a dependent adult and spent more time caregiving were more likely to also self-declare.

**Conclusions:**

Our results show low levels of agreement between the two main methods used to identify informal carers. Any assessment of current caregiving research or future means to collect caregiving information should pay particular attention to the identification method as it may only relate to certain carer groups.

**Supplementary Information:**

The online version contains supplementary material available at 10.1007/s40273-022-01136-8.

## Key Points

**Table:** 

There are two main methods to identify informal carers but little evidence on whether they identify the same individuals.
We show that declaration and activity-based approaches identify different numbers of carers and, though similar characteristics predict caregiving, their predictive effects differ in magnitude.
Careful consideration is needed on the method of identification when incorporating informal care in economic evaluation.

## Introduction

The inclusion of informal care in research studies first involves its conceptualisation followed by its measurement [1, 2]. The measurement stage can be split into two parts—identification and estimation of its extent. Identification determines who further caregiving-related information is collected from and is an integral part of the measurement stage. These are important first steps for studies that seek to value caregiving for an economic evaluation or examine the consequences of caregiving [3].

There is no formal consensus on how to identify whether an individual is an informal carer. As an estimated 10% of the England and Wales population, some 5.8 million people, declared they provided some informal care in the 2011 census [4], even small differences between identification methods would apply to a sizeable population.

The most common method of identifying carers is via self-declaration in specific questions such as those used in household or ageing surveys [5, 6]. An example caregiving question that is similar to household or ageing surveys is from the 2021 England and Wales census, which was worded as follows:

“Do you look after, or give any help or support to, anyone because they have long-term physical or mental health conditions or illnesses, or problems related to old age? Do not count anything you do as part of paid employment” [7]

However, individuals may not wish to declare themselves as a ‘carer’ due to public stigma, may not realise they are a carer or may view themselves under a different role, such as a spouse or son/daughter [8–10]. A further issue is the discrepancy between provider and recipient reports [11, 12]. An alternative method is to use time diaries in which respondents detail what they were doing throughout an entire day [13]. Time use surveys can identify carers indirectly by performance of caregiving activities. We refer to this as an activity-based method.

The quantitative literature on carer identification is limited in two key areas. First, there is no evidence on the degree to which different methods capture the caregiving population. Second, it is not clear whether and to what degree the factors associated with caregiving differ across identification methods. Studies that compare informal care across different methods only consider reported time between a self-declaration recall questionnaire and a time diary. Other work related to identification has only explored that of non-primary carers [14–16].

The literature regarding the measurement of informal care has focused on issues related to current means of measuring caregiving time [1, 17]. These issues include the omission of non-tangible activities that could be viewed as caregiving [17], the difficulty in separating normal day-to-day tasks from caregiving tasks and that accounting for multitasking is a considerable challenge [18]. Time diaries are often viewed as a means of addressing some of these issues. Caregiving studies that use time diary data in some cases do not compare with non-carers, but examine activity based on some form of prior self-declaration, for example through a carer group [18, 19].

Only one study by Freedman et al. [20] considers both declaration and activity as a means of identifying carers using the 2013 Disability and Use of Time supplement of the US Panel Study of Income and Dynamics. Out of 3505 respondents, 332 (9.4%) were self-declared carers and 179 (5.1%) were activity-defined carers. This may suggest that a higher proportion of individuals may self-declare than report caregiving activity within the diary window. However, the study did not report the extent of agreement.

We investigate whether, and to what degree, two approaches of informal carer identification produce different carer groups in terms of their number as well as their health and demographic characteristics. We are the first to compare self-declaration and activity-based methods for identifying carers. Using the 2014/2015 UK Time Use Survey, we first compare the factors associated with caregiving across declaration and activity-based methods. Second, we consider for each method what factors are associated with carers being identified by both declaration and their activity.

## Data and Methodology

The 2014/15 UK Time Use Survey (UKTUS) is one of the largest surveys of its kind to be carried out in the UK [21]. The survey is nationally representative of individuals and households in the UK. It contains three major components: a household questionnaire, an individual questionnaire and a time diary. The survey aimed to collect time diaries from everyone 8 years old and over in 5500 households in the UK and achieved a household response rate of 40% [22]. The time diary allowed individuals to record primary, secondary and tertiary activities in 10-min intervals across a 24-h period from 4 am to 4 am the subsequent day for one weekday and one weekend day. Reported activities in completed diaries are classified according to a list of activity codes [21]. We chose the UKTUS because there was a high likelihood we would identify a substantial number of activity-based carers given the diary window is over 2 days and the ability within the diary to record multitasking. These are important advantages over other time use surveys, for example, the American Time Use Survey (ATUS) covers only 1 day and does not allow for multitasking.

### Defining Caregiving

We adopted two approaches to identify an informal carer: (i) self-identification from a survey questionnaire and (ii) self-reported activity from a time diary where the respondent records their activity from 2 days.

The first was based upon the respondent self-declaring as a co-residential carer in the individual questionnaire. The co-residential question is worded:

Is there anyone living with you who is sick, disabled or elderly whom you look after or give special help to, other than in a professional capacity?

We excluded respondents that provide care to a recipient under 18 years old to be consistent with the activity-based method.

The second approach uses the record of activity information provided in the time diary. Respondents are asked to record all their activity for 2 days. From this record, we classified respondents as activity-based carers if they recorded spending any amount of time on at least one of the four caregiving-related activities as a primary, secondary or tertiary activity at any point during the two diary days. This caregiving definition covers four activities provided to an adult household member who is either a dependent (e.g. Alzheimic parent or permanently/semi-permanently disabled) or non-dependent (e.g. temporarily disabled or injured) which includes: (i) Unspecified help; (ii) Physical care; (iii) Accompanying; and (iv) Other specified help. Examples of the types of activities under these four activity groups are ‘Physical care of a disabled, sick or elderly adult; washing, cutting hair, massaging; mental help, information and advice; accompanying an adult to a doctor and visits to hospitals’ [22]. We based this choice of caregiving-related activities on the definition used by the Office for National Statistics for caregiving as part of their national household satellite accounts [23]. We included only caregiving activities performed for a co-resident to be consistent with the coverage of the self-declaration question.

### Empirical Strategy

We first present the number of individuals that are classified as neither declared nor activity carers; activity only carers; declared only carers; and declared and activity carers.

Second, we explore how factors associated with caregiving differ across self-declaration and activity-based methods of identification. We consider two binary outcomes where ${D}_{i}$ equals one if respondent *i* self-declares as a carer (and zero otherwise) and ${A}_{i}$ equals one if they perform caregiving-related activity in the diary (and zero otherwise). Some respondents classified as ‘non-activity identified’ by the activity-based method may be self-declared carers and some respondents classified as ‘non-self-declared’ by the declaration method may be activity-based carers. We jointly estimate these outcomes with a bivariate probit estimator [24]:

1 $${D}_{i}={\alpha }_{1}+ {\gamma }_{j}{X}_{i}+{u}_{i1}$$

2 $${A}_{i}={\alpha }_{2}+ {\partial }_{j}{X}_{i}+{u}_{i2}$$

where $\alpha$ are intercepts, ${X}_{i}$ is the vector of covariates, ${\gamma }_{j}$ and ${\partial }_{j}$ the associated coefficients for each variable *j*, and ${u}_{i}$ are error terms. We use the ‘biprobit’ STATA command, report the average marginal effects, report standard errors clustered by the primary sampling unit and report the correlation coefficient for the error terms. The primary sampling units consisted of the postcode sector in Great Britain and wards in Northern Ireland. We chose the bivariate probit estimator because it accounts for correlation in the error terms between outcome models and eases testing of coefficients across outcomes [25]. We tested the equality of the coefficients across the equations and report the Chi-squared statistic.

Our covariate selection was based on variables commonly used in the literature exploring the determinants of caregiving directly [26] or indirectly through matching methods [27–29]. We include age, age squared, gender, marital status (single/never married, married/cohabiting, widowed/divorced), labour force status (employed, unemployed/inactive), education (degree or higher education, A-level/secondary education/equivalent, vocational/other/no education), born in the UK, tenure (owning a house), having a long-standing health condition or disability expected to last longer than a year, self-assessed health (very good, good, fair, bad/very bad), and the numbers of adults and children in the household. We include total monthly household income only in a sensitivity analysis as this variable had substantially more missing values than the other covariates. We further report, as sensitivity checks, analyses using standard errors clustered by the household, the application of sample weights, estimation of the model with seemingly unrelated regression using OLS and with independent probit regressions.

Third, we compare different types of identified carers. This considers the agreement between declared and activity-identified carers in two models estimated independently with probit regressions:

3 $${DB}_{i}={\alpha }_{3}+ {\varnothing }_{j}{X}_{i}+{\mu }_{j}{CD}_{i}+{u}_{i3}$$

4 $${AB}_{i}={\alpha }_{4}+ {\omega }_{j}{X}_{i}+{\rho }_{j}{CA}_{i}+{u}_{i4}$$

where outcomes ${DB}_{i}$ and ${AB}_{i}$ equal one if the respondent is both an activity-identified and a declared carer. The outcome ${DB}_{i}$ equals zero if the respondent is a ‘declared only’ carer whereas outcome ${AB}_{i}$ equals zero if they are an ‘activity only’ carer. In other words, Eq. (3) identifies characteristics predicting performance of any caregiving activity among those who self-declare as a carer. Equation (4) is estimated among those who perform caregiving activities and identifies characteristics predicting those who also self-declare caregiving status.

We include additional caregiving-related covariates available from each method of identification. In the activity-based model we include the total (logged) caregiving time, the number of activities provided to a dependent and non-dependent recipient and whether the individual performs caregiving activities on both diary days (denoted ${CA}_{i}$). In the declaration-based model we include the relationship of the carer to the recipient (denoted ${CD}_{i}$) in five categories: spousal (spouse and co-habiting partner), parent (to a son/daughter including adopted, son-in-law/daughter-in-law), son/daughter (to a parent/guardian and step parent), other relative/non-relative (brother/sister including adopted, grandchild, other relative and other non-relative) and an additional category for those with more than one recipient.

For sensitivity analysis we used a multinomial logit regression where the outcome takes a value of one if the carer is activity only, two if the carer is declared only and three if the carer is both groups. We chose this estimation as a sensitivity analysis because, whilst it is a more granular comparison between carer groups, it prohibits the inclusion of caregiving-specific covariates that are only measured for self-declared or activity-identified carers. We also performed a check on the multinomial logit in the form of a Wald test of combining alternative outcomes to provide evidence for whether any of the outcomes should be combined.

### Sample Restrictions

We removed individuals that are under 18 years old, have at least one diary with more than 90 min of incomplete activity information, have incomplete information on any included covariate and provide care to anyone below 18 years old. The incomplete activity restriction is a recommended marker for diary quality [22]. These restrictions reduced the total number of individuals in the sample by 23.8% from 8274 to 6301 with the diary quality and respondent age restriction accounting for the majority of this sample loss (see Table A1 in the electronic supplementary material [ESM] for details on each restriction). We were only able to analyse co-residential carers as there was no extra-residential self-declaration question in the survey. The proportion of self-declared carers that were lost due to all the sample restrictions was 31.4%, whereas for activity carers this was lower at 15.2%. The proportion of self-declared and activity-based carers remained fairly constant with the raw sample without restrictions at 5.7% and 4.4%, respectively (see Table A1 in the ESM). Under 1% of respondents had only one diary day of recorded activity in all carer and non-carer groups, which suggests that activity carers are not fewer in number because they are more likely to complete only one diary (Table A1, see ESM). Our final sample contained 6301 individuals, of which 304 (4.8%) were activity-defined carers and 345 (5.5%) were self-declared carers.

## Results

### Informal Carer Sample Composition

A total of 91.35% of the sample did not identify as a co-residential carer with either activity or self-declared approaches (Table 1). Conditional on a respondent identifying as a carer via either of the two approaches (*n* = 545), 36.70% were activity only, 44.22% were declared only and 19.08% were identified by both methods.

**Table 1:** Sample composition by method of informal carer identification

	Non-carers	Activity-identified only	Self-declared only	Both
Individuals	5756	200	241	104
(% of the sample)	(91.35)	(3.17)	(3.82)	(1.65)
[% of all carers]	–	[36.70]	[44.22]	[19.08]

Table 2 presents detail on the type and amount of activities performed by the 304 activity-identified carers. Activity-identified carers spent, on average, 80.4 (SD 201.5) min on 1.2 (SD 0.5) caregiving activities across all diary days. An activity was performed for a non-dependent adult household member by 85.5% of activity-identified carers and 17.8% performed an activity for a dependent adult household member. The most common activity was ‘physical care of a non-dependent’ as 46.4% performed this activity. Care to dependents accounted for the largest time, with a mean value of 23.6 (SD 181.2) min across all diary days for ‘unspecified help to a dependent adult household member’.

**Table 2:** Summary statistics of caregiving activities among 304 activity-identified informal carers

	Activities over 2 diary days				
	Number of carers that perform this activity/ these activities (%)	Mean minutes for all carers (SD)	Mean minutes conditional on activity/ activities participation (SD)	Mean number of activities (SD)	Mean number of activities conditional on participation (SD)
Care to a non-dependent adult household member					
Unspecified help	34 (11.2%)	6.2 (26.2)	55.6 (59.1)	–	–
Physical care	141 (46.4%)	14.7 (34.6)	31.8 (45.2)	–	–
Accompanying	25 (8.2%)	5.1 (23.8)	61.6 (59.6)	–	–
Other specified help	89 (29.3%)	11.3 (28.1)	38.5 (40.6)	–	–
Total of care to non-dependents	260 (85.5%)	37.3 (54.9)	43.6 (57.0)	1.0 (0.5)	1.11 (0.3)
Care to a dependent adult household member					
Unspecified help	20 (6.6%)	23.6 (181.2)	358.5 (630.4)	–	–
Physical care	36 (11.8%)	16.2 (59.5)	136.9 (116.6)	–	–
Accompanying	6 (2.0%)	1.5 (16.4)	76.7 (96.7)	–	–
Other specified help	11 (3.6%)	1.7 (13.3)	48.2 (54.0)	–	–
Total of care to dependents	54 (17.8%)	43.1 (199.3)	242.4 (421.6)	0.24 (0.6)	1.35 (0.6)
Total of all care	304 (100%)	80.4 (201.5)	–	1.2 (0.5)	–

Activity information relates to primary, secondary and tertiary activities. The percentages provided are not mutually exclusive as carer giving can be provided to both a dependent and non-dependent adult household member

*SD* Standard deviation

### Factors Associated with Caregiving Including Non-carers

Activity-identified and self-declared caregivers compared with their respective non-carers are older, are more likely to be employed and are more likely to have a long-standing health condition compared with non-carers (Table 3). The two carer groups are similar in age but there are notable differences in marital status and employment.

**Table 3:** Summary statistics of carer and non-carer groups

Variable	Non-activity-identified group	Activity-identified carers	Non-self-declared group	Self-declared carers
Demographic and health variables				
Female	53.2%	60.9%	53.5%	54.5%
Age in years, mean (SD)	48.7 (17.9)	53.4 (16.6)	48.6 (17.8)	53.6 (17.7)
Marital status: Single or never married	20.4%	11.5%	19.9%	21.2%
Marital status: Married or cohabiting	63.1%	82.6%	63.6%	72.8%
Marital status: Divorced or widowed	16.4%	5.9%	16.5%	6.1%
LFS: Employed	39.0%	51.6%	38.5%	58.0%
Education: Degree or higher	44.0%	44.1%	44.5%	35.1%
Education: A-level or secondary	44.9%	41.1%	44.7%	45.5%
Education: Other	11.0%	14.8%	10.7%	19.4%
UK born	87.8%	87.2%	87.4%	93.3%
Owns house	71.1%	71.4%	71.6%	63.5%
# of Adults in household, mean (SD)	2.20 (1.00)	2.52 (0.95)	2.20 (1.00)	2.57 (0.95)
# of Children in household, mean (SD)	0.53 (0.92)	0.45 (0.83)	0.53 (0.92)	0.38 (0.92)
SAH: Very good	33.5%	26.3%	33.8%	21.7%
SAH: Good	42.8%	46.7%	43.1%	40.9%
SAH: Fair	17.5%	20.4%	17.0%	28.7%
SAH: Bad/very bad	6.2%	6.6%	6.0%	8.7%
Long-standing health condition	36.6%	45.1%	36.3%	49.0%
Individuals	5997	304	5956	345
% of the sample	95.2%	4.8%	94.5%	5.5%

Activity-identified carers and self-declared carers in this table are not mutually exclusive as 104 individuals are classified as both activity-identified and self-declared caregivers

*LFS* labour force status, *SAH* self-assessed health, *SD* standard deviation

We present the factors associated with caregiving identified from activity and declaration methods in Table 4. The statistically significant and positive correlation coefficient (*ρ*) indicates evidence in favour of estimating the two outcomes as a bivariate probit. The coefficients are further from zero for the self-declared compared with the activity-identified carer model. In particular, for age, marital status, the UK born indicator, the household ownership indicator and household composition. These coefficients, except for household composition, are statistically different across models as indicated by the test of equality from the Chi-squared statistic. Those who are married/cohabiting are 2.4% (*p* < 0.01) points more likely to be an activity-identified carer than those who are single/never married, whereas the equivalent association is − 1.9% (*p* > 0.1) points for self-declared carers.

**Table 4:** Marginal effects from a bivariate probit regression of the factors associated with two methods of identifying informal carers

	Pr (activity-identified carer)	Pr (self-declared carer)	Test of equality of coefficients (*χ*^2^)
Female	0.018*** (0.005)	0.007 (0.006)	3.322*
Age/100 (years)	0.249** (0.104)	0.401*** (0.111)	1.461
Age squared/100	− 0.171* (0.097)	− 0.268*** (0.102)	0.797
Marital status: Married/cohabiting	0.024*** (0.009)	− 0.019 (0.012)	10.689***
Marital status: Divorced/widowed	− 0.019** (0.008)	− 0.062*** (0.012)	10.515***
LFS: Employed	0.018*** (0.007)	0.026*** (0.008)	0.859
Education: Degree or higher	− 0.003 (0.009)	− 0.019* (0.010)	2.557
Education: A-level or secondary	− 0.011 (0.009)	− 0.017* (0.010)	1.058
UK born	0.003 (0.010)	0.042*** (0.012)	7.678***
Owns house	− 0.016** (0.007)	− 0.034*** (0.008)	3.942**
# of Adults in household	0.018*** (0.003)	0.024*** (0.004)	1.699
# of Children in household	0.000 (0.003)	0.002 (0.004)	0.028
SAH: Good	− 0.002 (0.012)	− 0.015 (0.013)	0.900
SAH: Fair	0.007 (0.011)	− 0.003 (0.012)	0.780
SAH: Bad/very bad	0.004 (0.011)	0.021 (0.013)	0.764
Long-standing health condition	0.009 (0.006)	0.005 (0.007)	0.309
Individuals	6304	6304	
Mean of the dependent variable	0.048	0.055	
*ρ*	0.579 (0.033)		
Wald test *ρ*=0	Chi2(1) = 179.228***		

The reported effects are average marginal effects

The reference category for marital status is single/never married, for LFS is unemployed/inactive, for education is vocational/other/no qualification and for SAH is very good health

The Chi-squared statistic is reported for the test of equality in coefficients and a further Chi-squared statistic for the correlation coefficient

Clustered standard errors at the primary sampling unit in parenthesis: *p < 0.1; **p < 0.05; ***p < 0.01

*LFS* labour force status, *SAH* self-assessed health

The probability of reporting as a carer increases with the log of household monthly income across both approaches and is presented in Appendix Table A2 (see ESM). The inclusion of income reduces the sample by 22.3% from 6301 to 4894 individuals. Other coefficients such as labour force status and education become closer to zero when income is included. Our results in Table 4 are similar across a range of sensitivity analyses that include standard errors clustered at the household level (Table A2, see ESM); the addition of individual sample weights (Table A2, see ESM); and with a seemingly unrelated regression estimated using OLS (Table A3, see ESM).

### Factors Associated with Caregiving Across Each Identification Method

Table 5 presents the summary statistics of carers who are only identified from activity, self-declaration and from both approaches. Those who are identified from both approaches are the oldest carer group on average and have the lowest proportion with very good self-assessed health relative to the other two carer groups. Carers that are identified by both approaches, relative to those identified by one approach, have the highest average total caregiving time at 170.79 (SD 325.05) min across all diary days and have the highest proportion of spousal carers at 72.3%. A plot of total time spent caregiving for ‘activity only’ carers and those identified with both methods shows that no ‘activity only’ carer reports above 220 min of total caregiving (Fig. A4, see ESM).

**Table 5:** Summary statistics of different carer groups

Variable	Activity only	Self-declared only	Activity and self-declared
Female	64.0%	54.6%	55.4%
Age in years, mean (SD)	49.5 (16.3)	50.3 (17.9)	61.3 (14.1)
Marital status: Single or never married	10.5%	24.6%	10.9%
Marital status: Married or cohabiting	83.0%	69.2%	84.2%
Marital status: Divorced or widowed	6.5%	6.2%	5.0%
LFS: Employed	40.0%	50.8%	74.3%
Education: Degree or higher	47.5%	33.8%	37.6%
Education: A-level or secondary	40.5%	47.1%	42.6%
Education: Other	12.0%	19.2%	19.8%
UK born	84.5%	93.8%	93.1%
Owns house	71.5%	60.0%	73.3%
# of Adults in household, mean (SD)	2.57 (0.99)	2.63 (0.97)	2.42 (0.90)
# of Children in household, mean (SD)	0.59 (0.88)	0.47 (1.00)	0.19 (0.66)
SAH: Very good	31.0%	23.8%	17.8%
SAH: Good	48.5%	40.0%	42.6%
SAH: Fair	15.0%	27.9%	30.7%
SAH: Bad/very bad	5.5%	8.3%	8.9%
Long-standing health condition	39.0%	45.4%	57.4%
Caregiving covariates			
Number of activities to a dependent, mean (SD)	0.030 (0.20)		0.66 (0.79)
Number of activities to a non-dependent, mean (SD)	1.050 (0.30)		0.74 (0.72)
Total caregiving time (min), mean (SD)	33.25 (38.50)		170.79 (325.05)
Caregiving on both days	13.5%		60.4%
Recipient: Spousal		50.8%	72.3%
Recipient: Parent		22.1%	11.9%
Recipient: Son/daughter		18.8%	9.9%
Recipient: Other		5.4%	5.0%
Carer has more than one recipient		2.9%	1.0%
Individuals	200	240^a^	101^b^
% of activity identified	66.4%		33.6%
% of self-declared identified		70.4%	29.6%

*LFS* labour force status, *SAH* self-assessed health, *SD* standard deviation

^a^Reduced from 241 to 240 due to incomplete information on the relationship between provider and recipient

^b^Reduced from 104 to 101 due to incomplete information on the relationship between provider and recipient

Table 6 presents models that show comparisons of the both (activity and self-declared) group with those that are only activity-identified and separately with those that are only self-declared carers. Age and those in employment relative to non-active labour market participants are associated with a higher probability of a carer both self-declaring and performing care-related activity. Among activity-identified carers, the number of activities provided to a dependent adult and the total caregiving time is positively (by 9.3% points for each 1% increase in caregiving minutes; *p* < 0.01) related to a carer also self-declaring. Those activity-identified carers who are married/co-habiting or divorced/widowed are less likely to also self-declare relative to single or never married carers. Self-declared carers are more likely to be an activity-identified carer if they are caring for a parent (by 4.7% points; *p* > 0.1) or some other relative/non-relative (by 25.5% points; *p* > 0.1); however, these association are not statistically different from zero at the 10% level.

**Table 6:** Marginal effects from two probit regressions on the factors associated with caregiving groups

	Activity-identified		Self-declared		
	= 1 both = 0 activity only	= 1 both = 0 activity only	= 1 both = 0 self-declared only	= 1 both = 0 self-declared only	
Caregiving covariates					
Number of activities to dependents		0.223** (0.110)			
Number of activities to non-dependents		− 0.032 (0.062)			
Log of total caregiving minutes		0.093*** (0.022)			
Provided care on 2 days		0.117** (0.057)			
Parent recipient				0.047 (0.109)	
Son/daughter recipient				− 0.038 (0.074)	
Other relative/non-relative recipient				0.252 (0.156)	
Carer has more than one recipient				− 0.114 (0.145)	
Demographic and health covariates					
Female	0.003 (0.048)	0.031 (0.037)	0.019 (0.047)	0.018 (0.047)	
Age/100 (years)	2.898** (1.146)	1.524* (0.871)	1.747* (0.956)	2.181** (0.957)	
Age squared/100	− 1.816* (1.013)	− 1.052 (0.772)	− 1.098 (0.855)	− 1.492* (0.866)	
Marital status: Married/cohabiting	− 0.232** (0.092)	− 0.199*** (0.077)	− 0.016 (0.094)	0.034 (0.113)	
Marital status: Divorced/widowed	− 0.340*** (0.119)	− 0.332*** (0.095)	− 0.085 (0.116)	− 0.082 (0.113)	
LFS: Employed	0.223*** (0.057)	0.126*** (0.045)	0.149*** (0.056)	0.145*** (0.056)	
Education: Degree or higher	0.101 (0.066)	0.053 (0.051)	0.108 (0.066)	0.108* (0.065)	
Education: A-level or secondary level	0.085 (0.064)	0.122** (0.047)	0.087 (0.059)	0.084 (0.059)	
UK born	0.094 (0.085)	0.076 (0.067)	0.017 (0.105)	0.015 (0.104)	
Owns house	− 0.077 (0.066)	0.013 (0.052)	0.032 (0.057)	0.031 (0.058)	
# of Adults in household	0.018 (0.025)	0.013 (0.020)	− 0.002 (0.029)	− 0.013 (0.030)	
# of Children in household	− 0.003 (0.040)	0.018 (0.027)	− 0.015 (0.034)	− 0.004 (0.035)	
SAH: Good	− 0.122 (0.119)	− 0.065 (0.101)	0.014 (0.106)	0.018 (0.106)	
SAH: Fair	− 0.111 (0.109)	− 0.064 (0.091)	0.065 (0.093)	0.070 (0.094)	
SAH: Bad/very bad	0.083 (0.112)	0.047 (0.092)	0.028 (0.090)	0.032 (0.091)	
Long standing health condition	− 0.022 (0.056)	0.034 (0.048)	0.036 (0.056)	0.043 (0.056)	
N	301	301	341^a^	341^a^	
Mean of dependent variable	0.34	0.34	0.30	0.30	
McFadden’s adjusted R-Squared	0.097	0.313	0.008		− 0.006

Both regressions are estimated with two independent probits. The reported effects are marginal effects

The reference category for marital status is single/never married, for LFS is unemployed/inactive, for education is vocational/other/no qualification, for SAH is very good health and for the provider-recipient relationship is spousal

Activity-identified and self-declared carer groups are not mutually exclusive. 107 individuals are classified as both activity-identified and self-declared caregivers

Clustered standard errors at the primary sampling unit in parentheses: *p < 0.1; **p < 0.05; ***p < 0.01

*LFS* labour force status, *SAH* self-assessed health

^a^Reduced from 345 to 341 due to incomplete information on the relationship between provider and recipient

The results of the multinomial logistic regression specification are shown in Table A6 (see ESM). Carers identified from activity and self-declared approaches are more likely to be employed rather than inactive in the labour market and less likely to be divorced/widowed than single/never married (Table A6). The Wald test of alternative outcomes rejects the null hypothesis that any combination of carer groups should be combined (Table A5, see ESM).

## Discussion

### Main Findings

There has been little consideration of who identifies as an informal carer using different identification approaches despite the impact that misclassification could have on informal care research. We find that 8.6% of respondents in our sample are identified as co-residential informal carers by at least one of the activity-based or self-declared approaches. This figure is similar to the 10% estimate which includes both co- and extra-residential carers in the England and Wales 2011 Census [7]. Given that only 5.5% of our sample are self-declared carers and the number of carers may have increased from 2011 to 2014/15, we conclude that self-declaration may underestimate the total extent of caregiving.

However, there is only moderate agreement between the two methods of carer identification as just 19.1% of 545 respondents identified by either method are identified by both methods. Furthermore, we show that carers identified by self-declaration differ more from non-carers in terms of their characteristics than carers identified by types of activities undertaken. For example, being born in the UK is more strongly related to self-declaration than activity-based identification. This may indicate cultural differences surrounding the self-declaration of a caregiving role where caregiving is seen as part of family life across generations. Another characteristic that varies by the method used to identify carers is marital status. Relative to those who are single/never married, married/cohabiting respondents are less likely to self-declare but more likely to be an activity-based carer, indicating that married/cohabiting couples may not view certain activities as caregiving. This result complements discussion in the caregiving literature that some carers may not find it straightforward to separate caregiving and non-caregiving activities [34]. Both the UK born and marital status variables highlight that individuals may be more likely to perform care-related activities in the context of a relationship, but not immediately identify as a caregiver.

For activity-identified carers, more activities provided to dependents, more time spent caregiving and provision of care activities on two diary days were all associated with a higher probability of also self-declaring as a carer. These results highlight that activity-based methods are likely to capture the most intensive self-declared caregivers, in terms of the dependency status of the recipient and the time burden of care. As the diary is only over 2 days, the activity approach will likely identify some of the most intensive daily caregivers as well as those that happen to perform caregiving on the specified diary days.

### Strengths and Limitations

This study is the first to compare the identification of informal carers based on two commonly used approaches. We exploited rich data on a respondent’s activity over 2 days which includes information on primary, secondary and tertiary activities for analysis. The UK Time Use Survey is well placed to identify activity-based carers as other time use surveys such as the American Time Use Survey only cover primary activities across 1 day. The health and socio-demographic characteristics from the individual questionnaire of the UK Time Use Survey permits analysis of specific factors related to identification by each approach. Furthermore, our results are robust to a variety of different specifications.

There remain several limitations with the present study. First, we were only able to analyse co-residential carers. Extra-residential caregiving may exhibit more overlap across approaches as care provided in a different household may be more distinguishable than care in the same household.

Second, additional factors associated with caregiving such as the availability of informal care in the respondent’s family was not captured in the survey. These characteristics are often used to explain variation in the probability of informal caregiving [27–29]. Other information on labour market status such as full-time or part-time employment was not possible to include in the analysis. As we find that those active in the labour market are more likely to identify as a carer relative to those inactive when considering non-carers as the reference group, it may be that part-time work rather than full-time work was driving the result. The large body of evidence on the labour market effects of caregiving may qualify this as a possibility [5]. A further possibility is a ‘healthy worker effect’ where people in better health are more able to combine employment and caregiving responsibilities [30].

Third, both identification approaches conceptualise caregiving differently, which may, in part, explain the low agreement. The time diary relies upon coders to classify caregiving tasks under the catalogue of activities based on the descriptions given by the respondent. The self-declaration question relies upon respondents to decide what activities constitute ‘looking after’ or ‘giving special help to’ a co-residing household member. In other words, self-declaration leaves the decision of what constitutes caregiving up to the respondent whereas activity-based identification is based on analysis of activities performed by the respondent. This may enable a broader set of activities and therefore more caregivers to be identified with the self-declaration method. A different conceptualisation of the self-declaration question with a task-based approach may be a route for future work as this version of a self-declaration question is used when assigning a monetary valuation to the time costs of informal care [31]. A related point is that caregiving may cover short intensive periods of time that may be missed by a diary. This may highlight the need for further caregiving questions in surveys that obtain information on the length of time an individual is or was a carer.

### Implications

Our results complement the qualitative literature on carer identity which finds lower rates of self-identification for spousal carers [32, 33] due to individuals not viewing themselves as a carer or not wishing to declare themselves as a carer even if they do [8–10]. We highlight the scale of this issue quantitatively, which has key implications as this group may be less likely to seek support or formal assistance. Therefore, in order to identify carer groups who do not self-declare, perhaps particularly at the early stages of caregiving, activity-based methods may offer an alternative.

This study has important implications for those performing an economic evaluation, survey methodologists and secondary data analysts. Specifically, when trying to recruit carers, a combination of screening questions based on self-declaration and whether an individual has performed a list of activities (if a time diary is not feasible) may identify a broader set of caregivers. Otherwise, surveys must consider which groups of carers may be missed and whether they are likely to cause bias in relation to the study or survey objectives. Current evidence on caregiving effects will therefore only be generalisable to specific carer groups. Future work could consider the consequences of each identification method on economic evaluations, for instance.

Carer policy and support that is tailored to the characteristics and circumstances of self-declared carers should be aware of other carer groups that may have different characteristics in terms of age; marital status; ethnicity; and home ownership. Otherwise, policy makers may not meet their desire to improve the lives of all carers. This is most apparent in health care settings where means to identify informal carers are limited and the early and appropriate identification of carers may help to improve carer health and support [34, 35].

Most activity-defined caregiving in our sample was care to non-dependents, which indicates temporary care for an injured family member. Activity-based methods may identify certain types of caregivers that would not be accounted for with declaration questions. Conversely, self-declared methods may identify more non-married carers than activity-based methods. A literature review on the challenges of measuring informal care time highlighted the identification of informal carers as a key issue relevant for both monetary and non-monetary economic evaluation methods [36]. In particular, informal care monetary and non-monetary valuation methods should tailor the identification approach to the type of caregiving intended to be analysed. For instance, if the research objective is to analyse spousal caregiving, then an activity-based approach would be preferable. Otherwise, if differences in characteristics across carer groups are reflective of differences in health and/or carer-related quality of life as well as reported caregiving time, this may affect the results of a valuation method.

## Conclusion

There has been little consideration of the methods to identify informal carers given the frequent capture of this type of information in surveys. Through a comparison, for the first time, of activity-based and self-declared carers, we show that these methods produce different samples of carers across a range of characteristics. These findings are of relevance to a range of audiences including those undertaking an economic evaluation, survey methodologists, secondary data analysts and policy makers. Any assessment of the current evidence on caregiving should pay particular attention to the identification method as it may only relate to certain carer groups. Future attempts to identify carers should aim to capture a wider range of carers.

## Supplementary Information

Below is the link to the electronic supplementary material.

Supplementary file1 (DOCX 147 KB)

## Declarations

### Data Availability

The study uses data from the 2014/15 UK Time Use Survey (UKTUS), which is available by application from the UK Data Service (https://ukdataservice.ac.uk/). The authors are not permitted to release the copy of the UKTUS used for the study
