# Supplementary material for: A Comparison of Methods for Identifying Informal Carers: Self-Declaration Versus a Time Diary
Source: Pharmacoeconomics. 2022 Apr 8;40(6):611–21. doi: 10.1007/s40273-022-01136-8 (PMC9130170; doi:10.1007/s40273-022-01136-8)
Supplement: Supplementary file 1 — Supplementary file1 (DOCX 147 KB) [file 40273_2022_1136_MOESM1_ESM.docx]

Table A1: Sample restrictions on the number of individuals

|  | Non-activity-identified group | Activity-identified group | Non-self-declared group | Non-self-declared group | Total |
| --- | --- | --- | --- | --- | --- |
| Initial sample | 7912 | 362 | 7755 | 503 | 8274 |
| Final sample (% with only one diary day) | 6046 (0.1%) | 304 (0.0%) | 5956 (0.1%) | 345 (0.1%) | 6301 (0.1%) |
|  |  |  |  |  |  |
| Restriction (% loss from initial sample): | | | |  |  |
| Child or poor-quality diary | 1689(21.3%) | 49 (13.5%) | 1640(21.1%) | 92 (18.3%) | 1738(21.0%) |
| Incomplete covariates | 177 (2.2%) | 6 (1.7%) | 159 (2.1%) | 14 (2.8%) | 183 (2.2%) |
| Care provided to a child | 0 (0.0%) | 3 (0.8%) | 0 (0.0%) | 52 (10.3%) | 52 (0.6%) |
| *Notes:* A Child is defined as an individual younger than 18 years old | | | | | |

| Table A2: Sensitivity analysis with the inclusion of income, standard errors clustered by household and the addition of sample weights estimated with a bivariate probit regression | | | | | | |  | |  |
| --- | --- | --- | --- | --- | --- | --- | --- | --- | --- |
|  | Inclusion of income | | Household clustered std errors | | | Sample Weights | | | |
|  | Pr(Activity) | Pr(Declared) | | Pr(Activity) | Pr(Declared) | Pr(Activity) | | Pr(Declared) | |
| Female | 0.020*** | 0.010 | | 0.018*** | 0.007 | 0.017*** | | 0.006 | |
|  | (0.006) | (0.006) | | (0.005) | (0.005) | (0.006) | | (0.006) | |
| Age/100 (years) | 0.313*** | 0.328** | | 0.249** | 0.401*** | 0.295** | | 0.477*** | |
|  | (0.110) | (0.128) | | (0.102) | (0.105) | (0.116) | | (0.122) | |
| Age squared/100 | -0.221** | -0.198* | | -0.171* | -0.268*** | -0.209* | | -0.321*** | |
|  | (0.106) | (0.118) | | (0.096) | (0.097) | (0.108) | | (0.112) | |
| Marital status: Married/Cohabiting | 0.018* | -0.008 | | 0.024*** | -0.019 | 0.017 | | -0.039** | |
|  | (0.010) | (0.013) | | (0.009) | (0.012) | (0.011) | | (0.016) | |
| Marital status: Divorced/Widowed | -0.022** | -0.053*** | | -0.019** | -0.062*** | -0.029*** | | -0.081*** | |
|  | (0.010) | (0.012) | | (0.009) | (0.012) | (0.010) | | (0.015) | |
| LFS: Employed | 0.012 | 0.018** | | 0.018*** | 0.026*** | 0.019*** | | 0.025*** | |
|  | (0.008) | (0.009) | | (0.007) | (0.007) | (0.007) | | (0.009) | |
| Education: Degree or Higher | -0.001 | -0.009 | | -0.003 | -0.019* | -0.002 | | -0.020* | |
|  | (0.011) | (0.012) | | (0.010) | (0.010) | (0.010) | | (0.011) | |
| Education: A-level or Secondary | -0.014 | -0.011 | | -0.011 | -0.017* | -0.011 | | -0.022** | |
|  | (0.010) | (0.011) | | (0.009) | (0.010) | (0.009) | | (0.011) | |
| UK born | 0.013 | 0.051*** | | 0.003 | 0.042*** | 0.004 | | 0.056*** | |
|  | (0.011) | (0.015) | | (0.009) | (0.011) | (0.010) | | (0.014) | |
| Owns house | -0.015** | -0.037*** | | -0.016** | -0.034*** | -0.015** | | -0.031*** | |
|  | (0.007) | (0.009) | | (0.007) | (0.008) | (0.007) | | (0.009) | |
| # of Adults in household | 0.023*** | 0.030*** | | 0.018*** | 0.024*** | 0.016*** | | 0.026*** | |
|  | (0.004) | (0.004) | | (0.003) | (0.003) | (0.003) | | (0.004) | |
| # of Children in household | 0.004 | -0.002 | | 0.000 | 0.002 | -0.000 | | 0.002 | |
|  | (0.003) | (0.005) | | (0.003) | (0.004) | (0.003) | | (0.005) | |
| SAH: Good | -0.003 | -0.008 | | -0.002 | -0.015 | -0.005 | | -0.021 | |
|  | (0.013) | (0.014) | | (0.012) | (0.014) | (0.013) | | (0.015) | |
| SAH: Fair | 0.004 | 0.001 | | 0.007 | -0.003 | 0.006 | | -0.006 | |
|  | (0.012) | (0.013) | | (0.011) | (0.013) | (0.012) | | (0.014) | |
| SAH: Bad/Very Bad | 0.000 | 0.025* | | 0.004 | 0.021 | 0.001 | | 0.022 | |
|  | (0.012) | (0.014) | | (0.011) | (0.014) | (0.012) | | (0.015) | |
| Long standing health condition | 0.013* | 0.007 | | 0.009 | 0.005 | 0.009 | | -0.002 | |
|  | (0.008) | (0.007) | | (0.006) | (0.007) | (0.007) | | (0.008) | |
| Log(income) | -0.006* | -0.011*** | |  |  |  | |  | |
|  | (0.003) | (0.004) | |  |  |  | |  | |
| Individuals | 4891 | 4891 | | 6301 | 6301 | 6295 | | 6295 | |
| Mean of the dependent variable | 0.47 | 0.56 | | 0.48 | 0.55 | 0.48 | | 0.55 | |
| ρ | 0.578  (0.038) |  | | 0.579  (0.033) |  | 0.589 (0.035) | |  | |
| Wald test ρ=0 | Chi2(1) = 136.97*** | | | Chi2(1) = 173.88*** | | Chi2(1) = 159.74*** | | | |

*Notes:* The reported effects are the average marginal effects. LFS denotes labour force status. SAH denoted self-assessed health. The reference category for marital status is single/never married, for LFS is unemployed/inactive, for education is vocational/other/no qualification, for SAH is Very Good health. Clustered standard errors at the primary sampling unit for the inclusion of income and sample weights models are reported in parenthesis: *p<0.1; **p<0.05; ***p<0.01

| Table A3: Factors associated with informal care across two definitions estimated with a seemingly unrelated regression using OLS | | | |  |  |
| --- | --- | --- | --- | --- | --- |
|  | Pr(Activity  -identified carer) | Pr(Self  -declared carer) | Test of Equality of  Coefficients ()  | |  |
| Female | 0.018*** | 0.006 | 3.322* | |  |
|  | (0.005) | (0.006) |  | |  |
| Age/100 (years) | 0.273*** | 0.417*** | 1.461 | |  |
|  | (0.091) | (0.106) |  | |  |
| Age squared/100 | -0.189** | -0.290*** | 0.797 | |  |
|  | (0.089) | (0.104) |  | |  |
| Marital status: Married/Cohabiting | 0.021** | -0.014 | 10.689*** | |  |
|  | (0.008) | (0.010) |  | |  |
| Marital status: Divorced/Widowed | -0.027*** | -0.066*** | 10.515*** | |  |
|  | (0.009) | (0.011) |  | |  |
| LFS: Employed | 0.021*** | 0.030*** | 0.859 | |  |
|  | (0.007) | (0.009) |  | |  |
| Education: Degree or Higher | -0.003 | -0.025** | 2.557 | |  |
|  | (0.010) | (0.012) |  | |  |
| Education: A-level or Secondary | -0.010 | -0.024** | 1.058 | |  |
|  | (0.010) | (0.012) |  | |  |
| UK born | 0.002 | 0.035*** | 7.678** | |  |
|  | (0.010) | (0.008) |  | |  |
| Owns house | -0.016** | -0.036*** | 3.942* | |  |
|  | (0.007) | (0.009) |  | |  |
| # of Adults in household | 0.018*** | 0.024*** | 1.699 | |  |
|  | (0.004) | (0.005) |  | |  |
| # of Children in household | -0.001 | -0.000 | 0.028 | |  |
|  | (0.003) | (0.004) |  | |  |
| SAH: Good | 0.004 | -0.013 | 0.900 | |  |
|  | (0.013) | (0.016) |  | |  |
| SAH: Fair | 0.012 | -0.003 | 0.780 | |  |
|  | (0.013) | (0.015) |  | |  |
| SAH: Bad/Very Bad | 0.008 | 0.024 | 0.764 | |  |
|  | (0.013) | (0.017) |  | |  |
| Long standing health condition | 0.010 | 0.005 | 0.309 | |  |
|  | (0.007) | (0.007) |  | |  |
| N | 6301 | 6301 |  | |  |
| Mean of the dependent variable | 0.048 | 0.062 |  | |  |
| *Notes:* LFS denotes labour force status. SAH denoted self-assessed health. The reference category for marital status is single/never married, for LFS is unemployed/inactive, for education is vocational/other/no qualification and for SAH is Very Good health. The chi-squared statistic is reported for the test of equality in coefficients. Clustered standard errors at the primary sampling unit in parenthesis: *p<0.1; **p<0.05; ***p<0.01 | | | | | |

Figure A4: Plot of total caregiving activity for activity only carers and those identified by both methods

Notes: Each bar indicates the number of informal carers

Table A5: Wald test for combining alternatives

| Outcome combination | Chi-sq (degrees of freedom) | P-value |
| --- | --- | --- |
| Activity & Declared | 51.094 (16) | p<0.001 |
| Activity & Both | 65.216 (16) | p<0.001 |
| Declared & Both | 40.830 (16) | P=0.001 |
|  |  |  |

| Table A6: Factors associated with three caregiving groups estimated with a multinomial logit specification | | | |
| --- | --- | --- | --- |
|  | Pr(Activity Only) | Pr(Declared Only) | Pr(Both) |
| Female | 0.058 | -0.048 | -0.010 |
|  | (0.038) | (0.042) | (0.033) |
| Age/100 (years) | -1.752** | -0.180 | 1.932*** |
|  | (0.857) | (0.801) | (0.668) |
| Age squared/100 | 1.077 | 0.208 | -1.285** |
|  | (0.809) | (0.751) | (0.586) |
| Marital status: Married/Cohabiting | 0.260*** | -0.147* | -0.113 |
|  | (0.054) | (0.080) | (0.074) |
| Marital status: Divorced/Widowed | 0.245** | -0.076 | -0.169* |
|  | (0.103) | (0.122) | (0.087) |
| LFS: Employed | -0.100** | -0.031 | 0.130*** |
|  | (0.049) | (0.049) | (0.042) |
| Education: Degree or Higher | 0.010 | -0.083 | 0.073* |
|  | (0.068) | (0.069) | (0.044) |
| Education: A-level or Secondary level | -0.024 | -0.030 | 0.055 |
|  | (0.066) | (0.066) | (0.039) |
| UK born | -0.178*** | 0.170** | 0.008 |
|  | (0.067) | (0.086) | (0.068) |
| Owns house | 0.124** | -0.094* | -0.030 |
|  | (0.053) | (0.054) | (0.040) |
| # of Adults in household | -0.029 | 0.020 | 0.009 |
|  | (0.023) | (0.025) | (0.019) |
| # of Children in household | -0.005 | 0.029 | -0.023 |
|  | (0.028) | (0.031) | (0.030) |
| SAH: Good | 0.089 | -0.067 | -0.021 |
|  | (0.098) | (0.110) | (0.069) |
| SAH: Fair | 0.060 | -0.079 | 0.020 |
|  | (0.088) | (0.098) | (0.064) |
| SAH: Bad/Very Bad | -0.098 | 0.048 | 0.049 |
|  | (0.086) | (0.096) | (0.063) |
| Long standing health condition | 0.027 | -0.034 | 0.008 |
|  | (0.049) | (0.054) | (0.038) |
| N | 545 | 545 | 545 |
| Mean of dependent variable | 0.37 | 0.44 | 0.19 |
| *Notes:* The reported coefficients are the average marginal effects. LFS denotes labour force status. SAH denoted self-assessed health. The reference category for marital status is single/never married, for LFS is unemployed/inactive, for education is vocational/other/no qualification and for SAH is very good. Clustered standard errors at the primary sampling unit in parenthesis: *p<0.1; **p<0.05; ***p<0.01 | | | |
